# Phosphoglycerate mutase 5 aggravates alcoholic liver disease through disrupting VDAC-1-dependent mitochondrial integrity

**DOI:** 10.7150/ijms.93171

**Published:** 2024-02-25

**Authors:** Tian Xia, Jiachi Yu, Ye Chen, Xing Chang, Miao Meng

**Affiliations:** 1 Chinese PLA General Hospital, Medical School of Chinese PLA, Beijing 100853, China.; 2 Department of Clinical Laboratory Medicine, The First Medical Centre, Medical School of Chinese PLA, Beijing, China.; 3 Guang'anmen Hospital, China Academy of Chinese Medical Sciences, Beijing, 100053, China.

**Keywords:** ALD, mitochondria, Pgam5, VDAC1

## Abstract

Alcoholic liver disease (ALD) poses a substantial global health challenge, with its pathogenesis deeply rooted in mitochondrial dysfunction. Our study explores the pivotal roles of Phosphoglycerate mutase family member 5 (Pgam5) and Voltage-Dependent Anion Channel 1 (VDAC1) in the progression of ALD, providing novel insights into their interplay and impact on mitochondrial integrity. We demonstrate that Pgam5 silencing preserves hepatocyte viability and attenuates ethanol-induced apoptosis, underscoring its detrimental role in exacerbating hepatocyte dysfunction. Pgam5's influence extends to the regulation of VDAC1 oligomerization, a key process in mitochondrial permeability transition pore (mPTP) opening, mitochondrial swelling, and apoptosis initiation. Notably, the inhibition of VDAC1 oligomerization through Pgam5 silencing or pharmacological intervention (VBIT-12) significantly preserves mitochondrial function, evident in the maintenance of mitochondrial membrane potential and reduced reactive oxygen species (ROS) production. *In vivo* experiments using hepatocyte-specific *Pgam5* knockout (*Pgam5^hKO^*) and control mice reveal that *Pgam5* deficiency mitigates ethanol-induced liver histopathology, inflammation, lipid peroxidation, and metabolic disorder, further supporting its role in ALD progression. Our findings highlight the critical involvement of Pgam5 and VDAC1 in mitochondrial dysfunction in ALD, suggesting potential therapeutic targets. While promising, these findings necessitate further research, including human studies, to validate their clinical applicability and explore broader implications in liver diseases. Overall, our study provides a significant advancement in understanding ALD pathophysiology, paving the way for novel therapeutic strategies targeting mitochondrial pathways in ALD.

## Introduction

Alcoholic liver disease (ALD) represents a significant global health burden, characterized by a spectrum of liver pathology ranging from simple steatosis to cirrhosis and hepatocellular carcinoma 1. The disease's etiology is intricately linked to chronic alcohol consumption, though individual susceptibility varies widely, suggesting the influence of genetic and environmental factors 2, 3. Epidemiologically, ALD is a leading cause of liver-related morbidity and mortality worldwide, with prevalence rates closely mirroring patterns of alcohol consumption in different populations 4, 5. Recent trends indicate an alarming increase in ALD cases, particularly in younger demographics and women, underscoring the shifting patterns of alcohol use. The pathogenesis of ALD involves complex interactions between metabolic derangements induced by chronic alcohol exposure and immune responses 2, 6, 7. Alcohol metabolism in the liver generates reactive oxygen species and acetaldehyde, contributing to oxidative stress and inflammation, which are central to the progression of ALD 8, 9. Management of ALD hinges on abstinence from alcohol, which has been shown to halt or even reverse liver damage in early stages 8, 9. Nutritional support and management of alcohol withdrawal are also crucial aspects of treatment 10, 11. Pharmacologically, therapies targeting the inflammatory cascade and oxidative stress are under investigation, but currently, there is no FDA-approved specific treatment for ALD. Therefore, exploring the molecular mechanisms underlying ALD will open a new therapeutic window for the patients with ALD.

Mitochondria play a crucial role in the pathophysiology of ALD 12, 13. The impact of alcohol on mitochondrial function is multifaceted, contributing significantly to the development and progression of ALD 14, 15. Central to this process is the disruption of mitochondrial oxidative phosphorylation. Alcohol metabolism in the liver leads to the accumulation of NADH, shifting the redox state and interfering with the mitochondrial electron transport chain 13, 14. This disruption results in impaired ATP production and enhances the generation of reactive oxygen species (ROS), which are pivotal in inducing oxidative stress and subsequent hepatocellular damage. Moreover, alcohol metabolism produces acetaldehyde, a highly reactive and toxic compound 16-18. Acetaldehyde forms adducts with mitochondrial proteins and DNA, further impairing mitochondrial function and integrity. This damage is compounded by the depletion of glutathione, a key antioxidant, exacerbating oxidative stress and mitochondrial dysfunction 19, 20. Another significant aspect is the effect of alcohol on mitochondrial dynamics, including the processes of fission and fusion. Chronic alcohol exposure disrupts these dynamics, leading to mitochondrial fragmentation, a feature commonly observed in ALD 21, 22. These morphological changes are not merely a consequence of liver injury but actively contribute to the progression of ALD. Mitochondria are also involved in the regulation of apoptosis, and alcohol-induced mitochondrial dysfunction can trigger apoptotic pathways in hepatocytes 23, 24. This process is mediated through the release of pro-apoptotic factors like cytochrome c, further contributing to liver injury. In summary, the role of mitochondria in ALD is complex and involves a cascade of events triggered by alcohol metabolism. These include oxidative stress, impaired bioenergetics, mitochondrial DNA and protein damage, disrupted mitochondrial dynamics, and the induction of apoptosis. However, the upstream mediator of mitochondrial integrity has not been fully understood.

Phosphoglycerate mutase family member 5 (Pgam5), is an atypical mitochondrial phosphatase that has garnered significant attention in the field of mitochondrial quality control 25, 26. This enzyme, localized to the mitochondrial inner membrane, plays a pivotal role in modulating mitochondrial dynamics, including fission and fusion processes 27, 28. Pgam5's unique structural characteristics, including a lack of similarity to conventional phosphoglycerate mutases, distinguish it functionally and biochemically 29-31. Emerging research has elucidated Pgam5's involvement in a variety of cellular processes beyond mitochondrial dynamics. These include apoptosis, necroptosis, and the regulation of mitochondrial morphology 32-34. Additionally, Pgam5 interacts with several key proteins, such as Parkin and PINK1, which are integral to mitochondrial quality control mechanisms. Recent studies have reported that Pgam5 affects liver cancer proliferation 35 and participates into the hepatic inflammation 36. Besides, non-alcoholic steatohepatitis is also under the control of Pgam5 in a high fat/high fructose diet-treated mouse model 29. During hepatic ischemia reperfusion injury, Pgam5 is ubiquitinated and contributed to the activation of ASK1 and JNK pathway, resulting into the activation of hepatocyte apoptosis 37. In the present study, we asked whether Pgam5 promoted ALDH through affecting mitochondrial integrity.

## Methods

The experimental procedures in this study followed approved guidelines and protocols from the Institutional Animal Care and Use Committee of Southern University of Science and Technology (protocol WDSKY0201408). Mice were housed in pathogen-free conditions with a 12-hour light/dark schedule, and had unrestricted access to water and chow diet. Wild type (WT) C57BL/6J mice were obtained from Shanghai Laboratory Animal Company. *Pgam5^flox^* mice were crossed with *Alb^Cre+^* mice to generate hepatocyte-specific *Pgam5* knockout (*Pgam5^hKO^*) mice. To establish an *in vivo* ALD model, 8-week-old mice (WT, *Pgam5^flox^,* and *Pgam5^hKO^*) were acclimated to a Lieber-DeCarli liquid control diet (F1259SP; Bio-Serv, Flemington, NJ, USA) for 5 days, then pair-fed either the same control diet or a 5% ethanol-containing diet (F1258SP; Bio-Serv) for 8 weeks. On the final day, mice received maltose dextran (control; 9 g/kg) or ethanol (5 g/kg) and were sacrificed after 8 hours. Tissues were harvested promptly and either frozen in liquid nitrogen or fixed in paraformaldehyde for subsequent analysis.

### Histopathology analysis

Histopathological analysis was carried out on liver tissues that had been fixed in a 10% formaldehyde solution for over 24 hours. To visualize the lipid accumulation pattern, paraffin-embedded tissues were subjected to hematoxylin-eosin (H&E) staining. Standard techniques were employed for Masson staining to detect liver fibrosis. The histological characteristics were subsequently observed and documented using a light microscope (ECLIPSE 80i, Nikon, Tokyo, Japan).

### Measurement of mitochondrial enzymatic activities

The activities of mitochondrial complexes were evaluated using well-established protocols. Specifically, the activity of complex I was determined by measuring the colorimetric changes at a wavelength of 340nm during the oxidation of NADH. Similarly, the activity of complex III was assessed by analyzing the decyl-ubiquinol cytochrome c oxidoreductase measurements at a wavelength of 550nm.

### Cell viability assay

Cells were plated in a 96-well plate at a density of 2 × 10³ cells per well (100 µl). After a 12-hour incubation period, the cells were treated with EtOH. Cell viability was determined using the cell counting kit-8 (CCK-8; Bimake, Shanghai, China), and the absorbance was measured at 450 nm using the BioTek Synergy HTX multi-mode reader. The MTT assay was employed to assess cell viability, following previously described methods 38.

### qPCR Analysis

For qPCR analysis, liver tissue or cell samples were lysed with TRIzol reagent (Ambion/Life Technologies, Naugatuck, CT) and homogenized using a rotor-stator homogenizer (Polytron MR 2100 set at power 27, Kinematika AG, Switzerland). RNA isolation was performed according to the TRIzol protocol, and 500 ng of RNA was reverse transcribed (RT) using SCRIPT (Jena Bioscience, Germany). Quantitative PCR (qPCR) was carried out on a StepOne Real-Time PCR System (Thermo-Fisher Scientific, Waltham, MA) using SYBR Green Supermix (Bio-Rad, Hercules, CA) 39.

### Cell culture and treatment

Human L02 hepatocytes and primary hepatocytes were cultured in DMEM supplemented with 10% FBS and 1% penicillin-streptomycin at 37°C with 5% CO2 in a humidified incubator provided by Thermo Fisher Scientific 40. The L02 cell line was acquired from the Type Culture Collection of the Chinese Academy of Sciences in Shanghai, China. To create an *in vitro* model of alcoholic liver disease, the cells were subjected to treatment with ethanol for 48 h at a concentration of 100 mM based on our previous research 41. VBIT-12 (Selleck, #S8936, 10μm) was used to treat L02 cells 6 hours before EtOH treatment.

### Detection of serum and liver biochemistry

Serum levels of triglycerides (TGs) and total cholesterol (TC) were measured using an ADVIA 2400 Chemistry System analyzer (Siemens, Tarrytown, NY, USA) as per the manufacturer's instructions. The activity of liver-associated enzymes, alanine aminotransferase (ALT) and aspartate aminotransferase (AST), was also estimated using the same analyzer. In order to measure TG and TC levels in liver tissues and cells, commercial kits (290-63701 for TGs, 294-65801 for TC; Wako, Tokyo, Japan) were utilized following the manufacturer's instructions.

### Mitochondrial potential and mitochondrial ROS measurement

Mitochondrial membrane potential was assessed using the JC-1 probe (Mitochondrial Membrane Potential Assay Kit, #ab113850, Abcam), following our previously described protocol 42. Briefly, cells were rinsed with PBS and then incubated with the JC-1 probe for 15 minutes. Intracellular reactive oxygen species (ROS) were detected using MitoSOXTM Red (Invitrogen), as per the manufacturer's instructions. Cells were incubated with 5 uM MitoSOX in Hank's balanced salt solution at 37°C for 25 minutes, followed by three washes with HBSS solution. Qualitative fluorescence measurements were obtained using confocal microscopy, with excitation at 510 nm and emission collected at 580 nm. The fluorescence intensity was analyzed using ImageJ software.

### Transient transfection of siRNA

L02 cells were transiently transfected with siRNA using Lipofectamine RNAiMAX (Life Technologies) according to the manufacturer's instructions. Specifically, siRNA targeting Pgam5 (si-Pgam5) at a final concentration of 10nM and nonsilencing siRNAs ("scrambled" si-Control) at a final concentration of 10nM were obtained from Dharmacon. Briefly, cells were seeded in six-well plates or 10-cm dishes at a confluency of 50 to 60%. The following day, Lipofectamine RNAiMAX (Thermo scientific, 13778150) containing siRNA was used for transfection, and after 6 hours, the media was replaced with DMEM supplemented with 10% FBS. After an additional 24 hours, the cells were serum-starved with either DMEM media containing 0.5% or 2.5% FBS for another 24 hours before drug treatment.

### Statistical analyses

Data are expressed as mean ± SEM (standard error of the mean). Statistical analyses were conducted using GraphPad Prism 9 (GraphPad Software Inc, USA). The normal distribution of all sample sets was confirmed using the Shapiro-Wilk normality test. To compare two groups, a two-tailed unpaired Student t-test was employed. When comparing multiple groups to a single control, one-way analysis of variance (ANOVA) followed by Dunnett's multiple comparisons test was used. For comparisons among multiple groups, one-way ANOVA followed by Tukey's multiple comparisons test was utilized. In cases where there were two independent variables, two-way ANOVA followed by Tukey's multiple comparisons test was applied. The Mantel-Cox test was employed for survival curves. The value of n represents the number of independent experiments or mice per group. Statistical significance was defined as p < 0.05. Representative images were selected to best represent the observed conditions, in conjunction with the mean values of each condition.

## Results

### Attenuation of Ethanol-Induced Hepatocyte Dysfunction by Pgam5 Silencing

**Figure 1:**
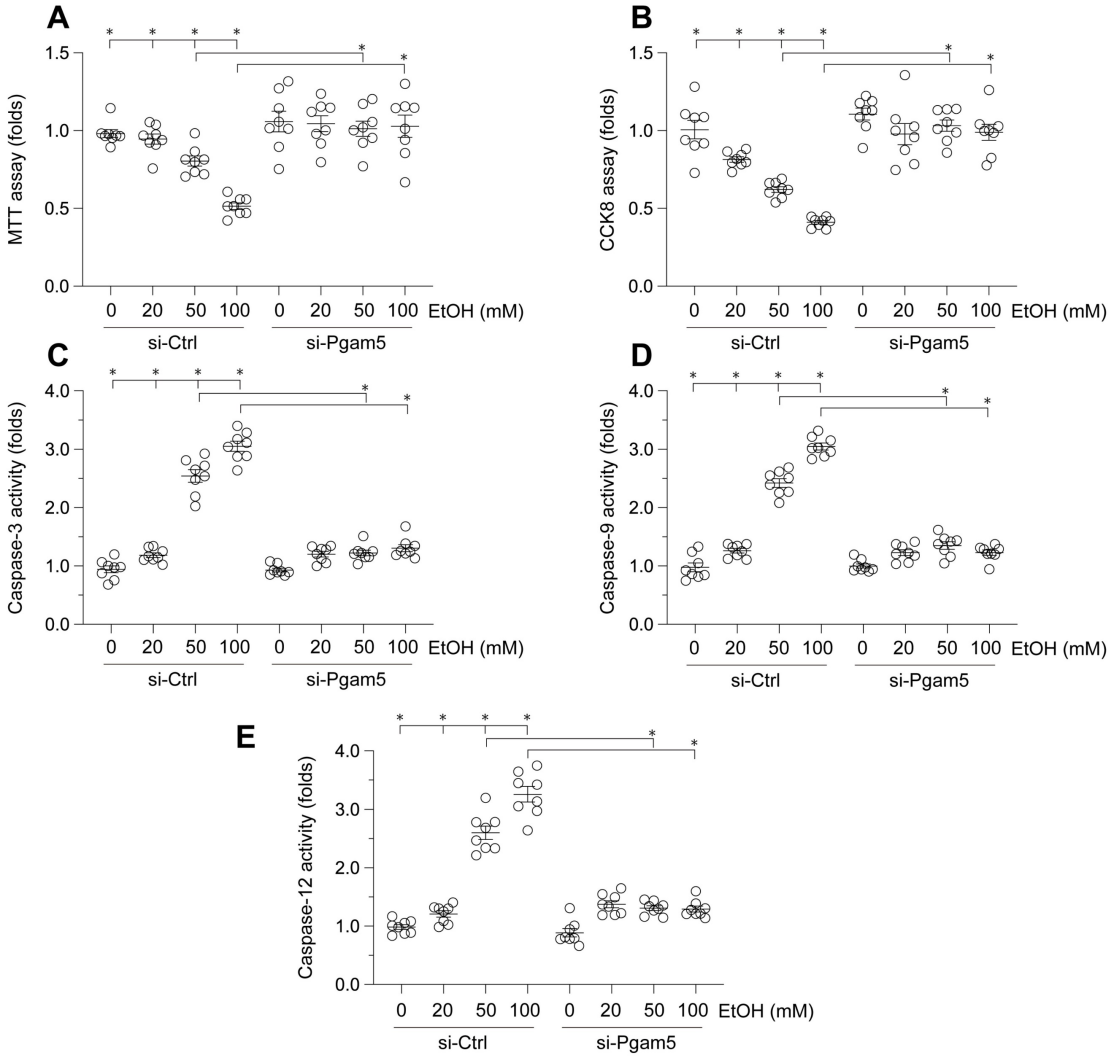
** Attenuation of Ethanol-Induced Hepatocyte Dysfunction by Pgam5 Silencing. A.** Cell viability was determined by MTT assay. **B.** CCK-8 assay was used to evaluate the cell viability. **C-E.** ELISA kits were used to evaluate the activities of caspase-3, caspase-9 and caspase-12. *p<0.05.

To elucidate the impact of Pgam5 on ethanol (EtOH)-mediated hepatocyte dysfunction, L02 cells were incubated with varying concentrations of EtOH. Cell viability was assessed via MTT and CCK-8 assays. As depicted in Figure 1A-B, EtOH treatment significantly reduced cell viability. Notably, Pgam5 silencing preserved hepatocyte viability in the presence of EtOH (Figure 1A-B). Given the established role of hepatocyte apoptosis in EtOH-induced pathology, we employed ELISA assays to evaluate apoptotic changes. EtOH exposure markedly increased caspase-3 activity, a response mitigated by Pgam5 deletion (Figure 1C). Additionally, activities of caspase-9 and caspase-12 were also elevated following EtOH exposure, but were attenuated by Pgam5 silencing (Figure 1D-E). Collectively, these findings corroborate that Pgam5 silencing diminishes EtOH-induced apoptosis in hepatocytes.

### Preservation of Mitochondrial Function in EtOH-Treated Hepatocytes by Pgam5 Silencing

**Figure 2:**
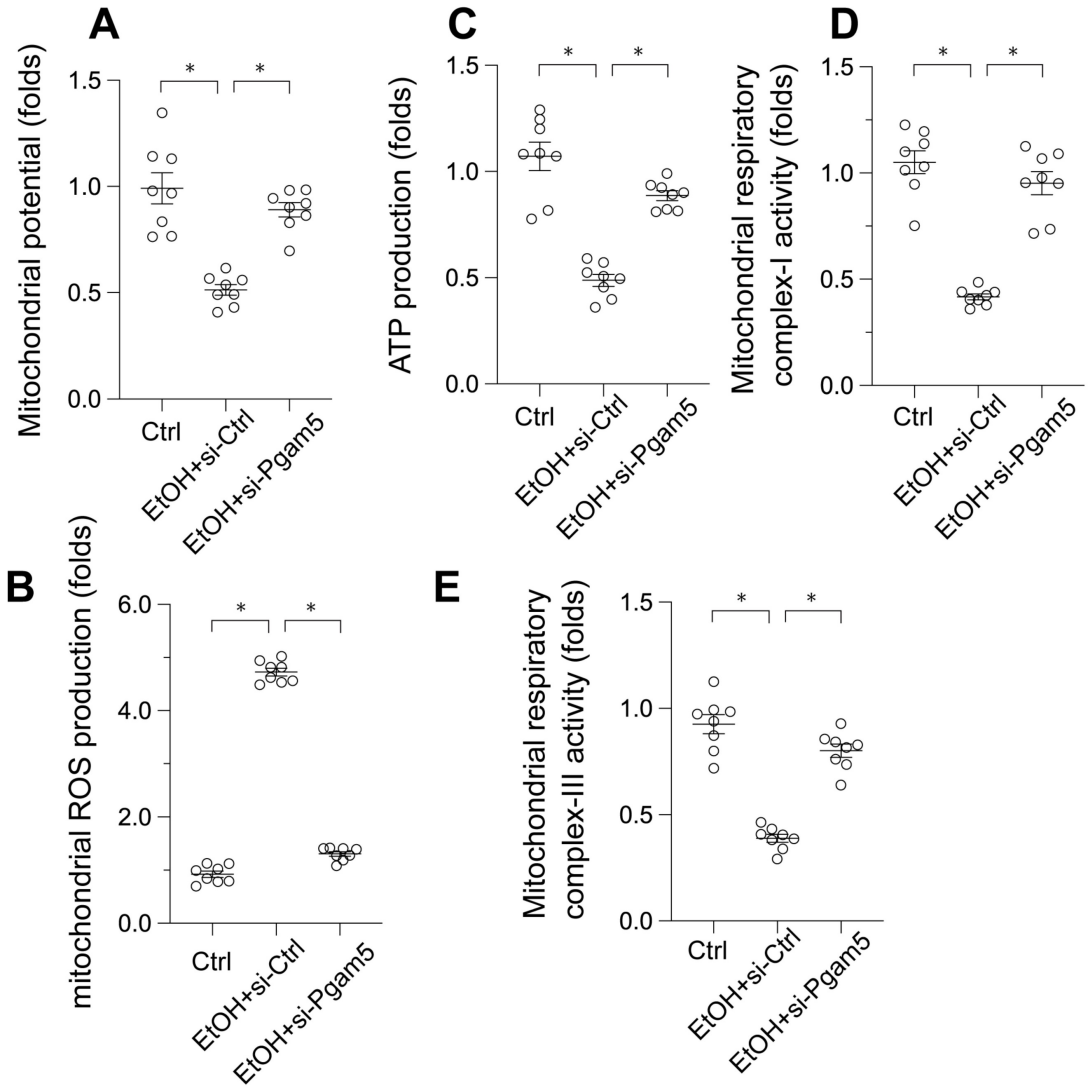
** Preservation of Mitochondrial Function in EtOH-Treated Hepatocytes by Pgam5 Silencing. A.** mitochondrial potential was measured by JC-1 probe. **B.** Mitochondrial ROS production was analyzed by immunofluorescence.** C.** ATP production was measured by an ELISA kit. **D-E.** The activities of mitochondrial respiratory complex I and III were determined by ELISA kit. *p<0.05.

Given the pivotal role of mitochondrial dysfunction in EtOH-induced hepatocyte apoptosis, we next examined mitochondrial function alterations. EtOH exposure led to a decrease in mitochondrial membrane potential, which was reversed by Pgam5 silencing (Figure 2A). Moreover, mitochondrial reactive oxygen species (ROS) production, initially augmented by EtOH, was mitigated following the loss of Pgam5 (Figure 2B). Similarly, mitochondrial ATP production, diminished in the presence of EtOH, remained unaffected in hepatocytes transfected with siRNA against Pgam5 (si-Pgam5) (Figure 2C). Furthermore, the activities of mitochondrial respiratory complexes I and III, inhibited by EtOH, were maintained in the absence of Pgam5 (Figure 2D-E). In summary, our results demonstrate that Pgam5 silencing effectively counteracts EtOH-induced mitochondrial dysfunction in hepatocytes *in vitro*.

### Pgam5 Silencing Reduces VDAC1 Oligomerization in Ethanol-Treated Hepatocytes

**Figure 3:**
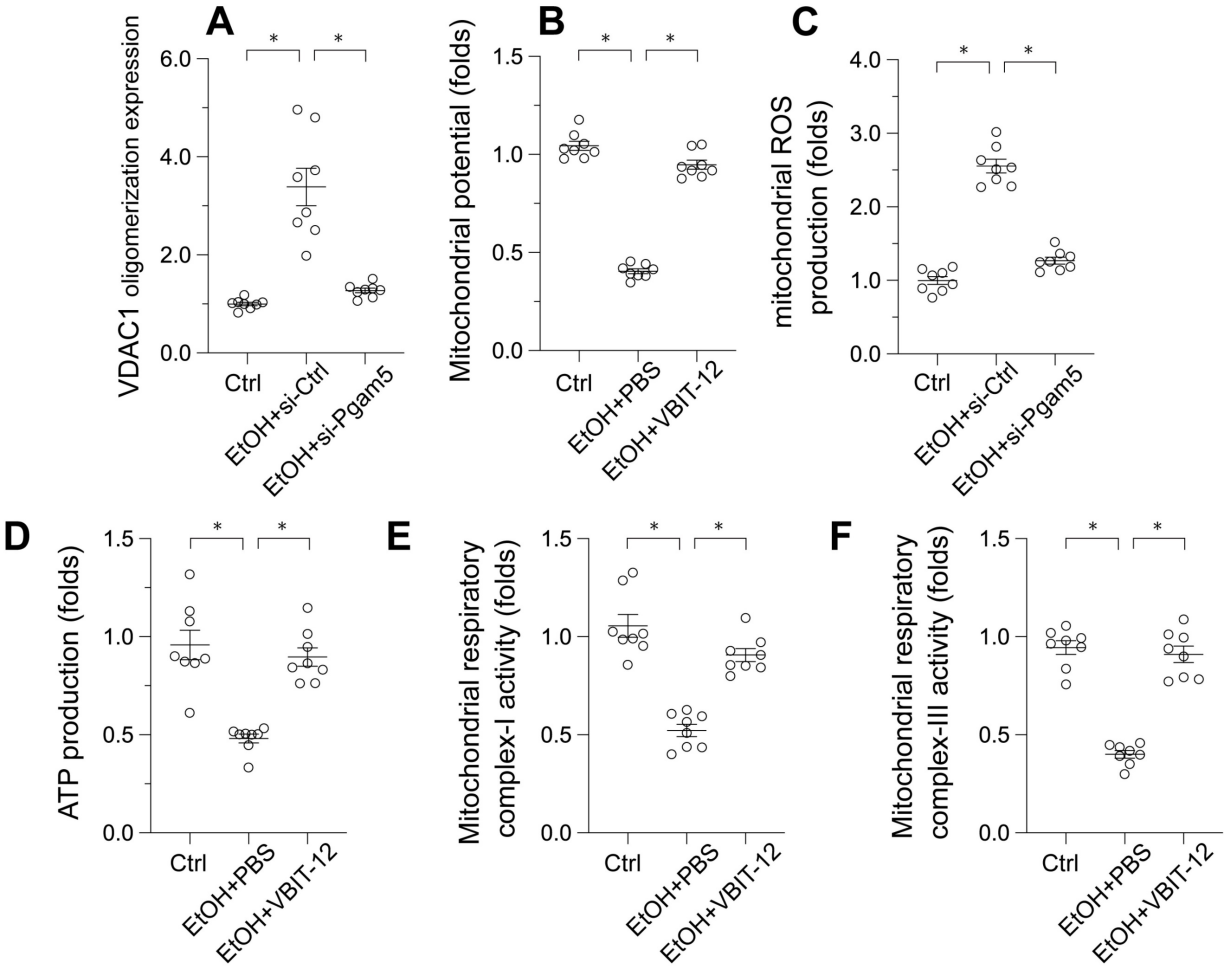
** Pgam5 Silencing Reduces VDAC1 Oligomerization in Ethanol-Treated Hepatocytes. A.** Western blots was used to analyze the protein expression of VDAC1 oligomerization.** B.** L02 cells were treated with VBIT-12 to inhibit the VDAC1 oligomerization and then mitochondrial potential was measured by JC-1 probe. **C.** L02 cells were treated with VBIT-12 to inhibit the VDAC1 oligomerization and then mitochondrial ROS production was analyzed by immunofluorescence. **D.** L02 cells were treated with VBIT-12 to inhibit the VDAC1 oligomerization and then ATP production was measured by an ELISA kit. **E-F.** L02 cells were treated with VBIT-12 to inhibit the VDAC1 oligomerization and then the activities of mitochondrial respiratory complex I and III were determined by ELISA kit. *p<0.05.

Voltage-Dependent Anion Channel 1 (VDAC1) has emerged as a critical regulator of mitochondrial integrity. Its oligomerization has been linked to mitochondrial permeability transition pore (mPTP) opening, mitochondrial swelling, and the initiation of apoptosis. To investigate the influence of ethanol (EtOH) on VDAC1 oligomerization, Western blot analyses were conducted. Following EtOH exposure, a pronounced increase in VDAC1 oligomerization was observed, a process that was inhibited by Pgam5 deletion (Figure 3A). This suggests Pgam5's role in VDAC1 oligomerization in alcohol-induced hepatocyte injury (Figure 3A). To determine the direct impact of VDAC1 oligomerization on mitochondrial dysfunction, L02 cells were treated with VBIT-12, an inhibitor of VDAC1 oligomerization, and reassessed for mitochondrial function. Figure 3B shows that VBIT-12 treatment resulted in increased mitochondrial membrane potential and reduced EtOH-induced mitochondrial ROS production (Figure 3C). Similarly, ATP production, which was inhibited by EtOH treatment, was improved by VBIT-12 treatment (Figure 3D). Additionally, mitochondrial respiratory complex activities, suppressed by EtOH, were restored to near-normal levels with VBIT-12 treatment (Figure 3E-F). These results collectively demonstrate that inhibiting VDAC1 oligomerization offers protection against EtOH-induced mitochondrial damage in hepatocytes.

### Pgam5 Deletion Mitigates Alcohol-Induced Liver Histopathology and Dysfunction

**Figure 4:**
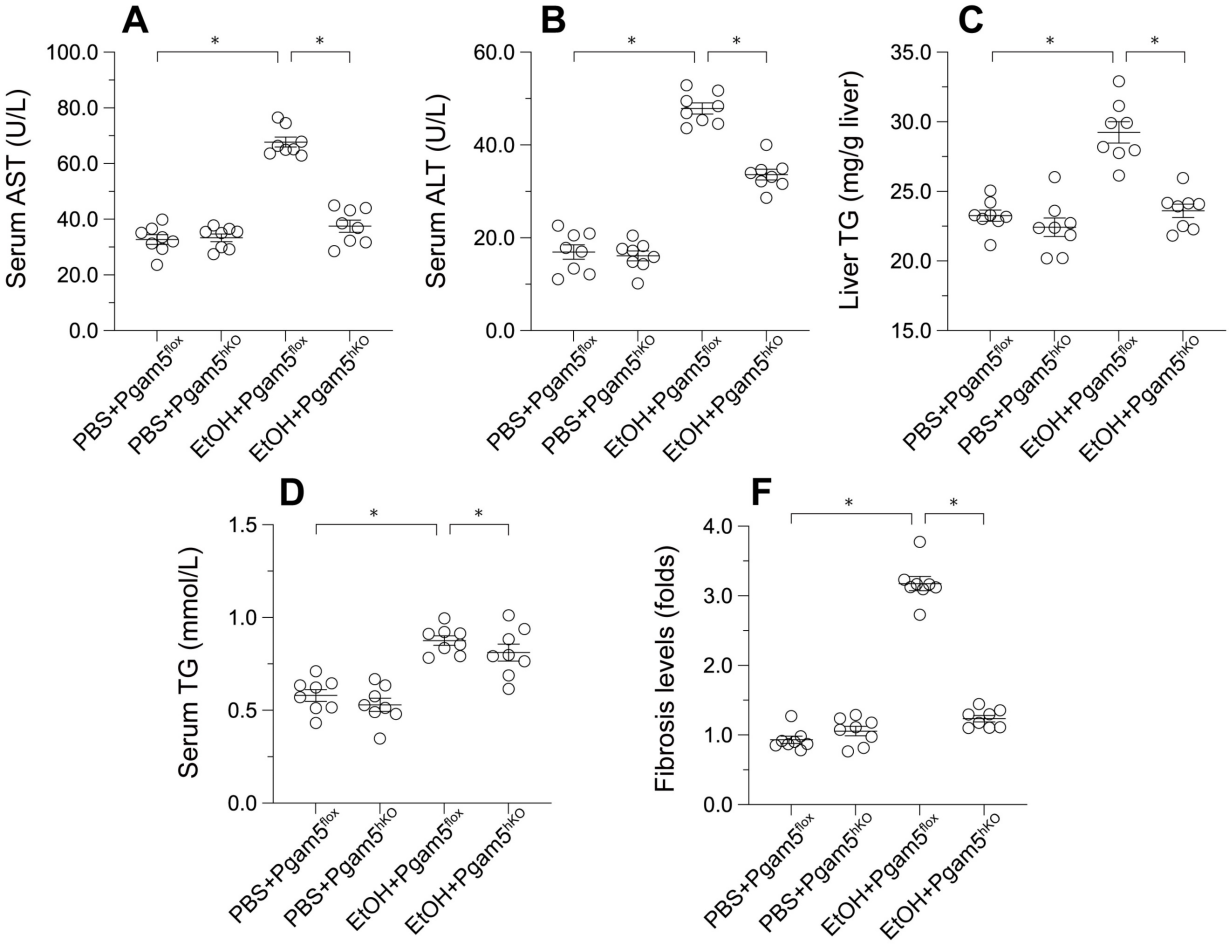
** Pgam5 Deletion Mitigates Alcohol-Induced Liver Histopathology and Dysfunction.** *Pgam5*-knock out (*Pgam5^hKO^*), and *Pgam5^f/f^* mice were fed with the liquid control (pair-fed) or ethanol diet (ethanol-fed) (5% ethanol) for 8 weeks. **A-B.** Serum ALT and AST was determined by ELISA. **C-D.** Serum TG and liver TG in *Pgam5^hKO^* and *Pgam5^f/f^* mice was measured by ELISA. **E.** Masson staining was used to display the hepatic fibrosis in *Pgam5^hKO^* and *Pgam5^f/f^* mice after exposure to EtOH. *p<0.05.

To explore Pgam5's role *in vivo*, hepatocyte-specific *Pgam5* knockout (*Pgam5^hKO^*) and *Pgam5^f/f^* control mice were subjected to a control or a 5% ethanol-containing liquid diet for 8 weeks. In *Pgam5^f/f^* mice, ethanol intake elevated serum alanine aminotransferase (ALT) and aspartate aminotransferase (AST) levels, indicators of liver injury, which were ameliorated in *Pgam5^hKO^* mice (Figures 4A-B). Ethanol also increased serum and liver triglyceride (TG) concentrations in male *Pgam5^f/f^* mice, an effect less pronounced in *Pgam5^hKO^* mice (Figures 4C-D). Additionally, mild hepatic fibrosis observed in *Pgam5^f/f^* mice post-EtOH treatment was absent in *Pgam5^hKO^* mice (Figures 4E), further underscoring Pgam5's protective role against alcohol-induced structural and functional liver abnormalities.

### Pgam5 Deletion Alleviates Alcohol-Induced Hepatic Inflammation, Lipid Peroxidation, and Metabolic Disorder

**Figure 5:**
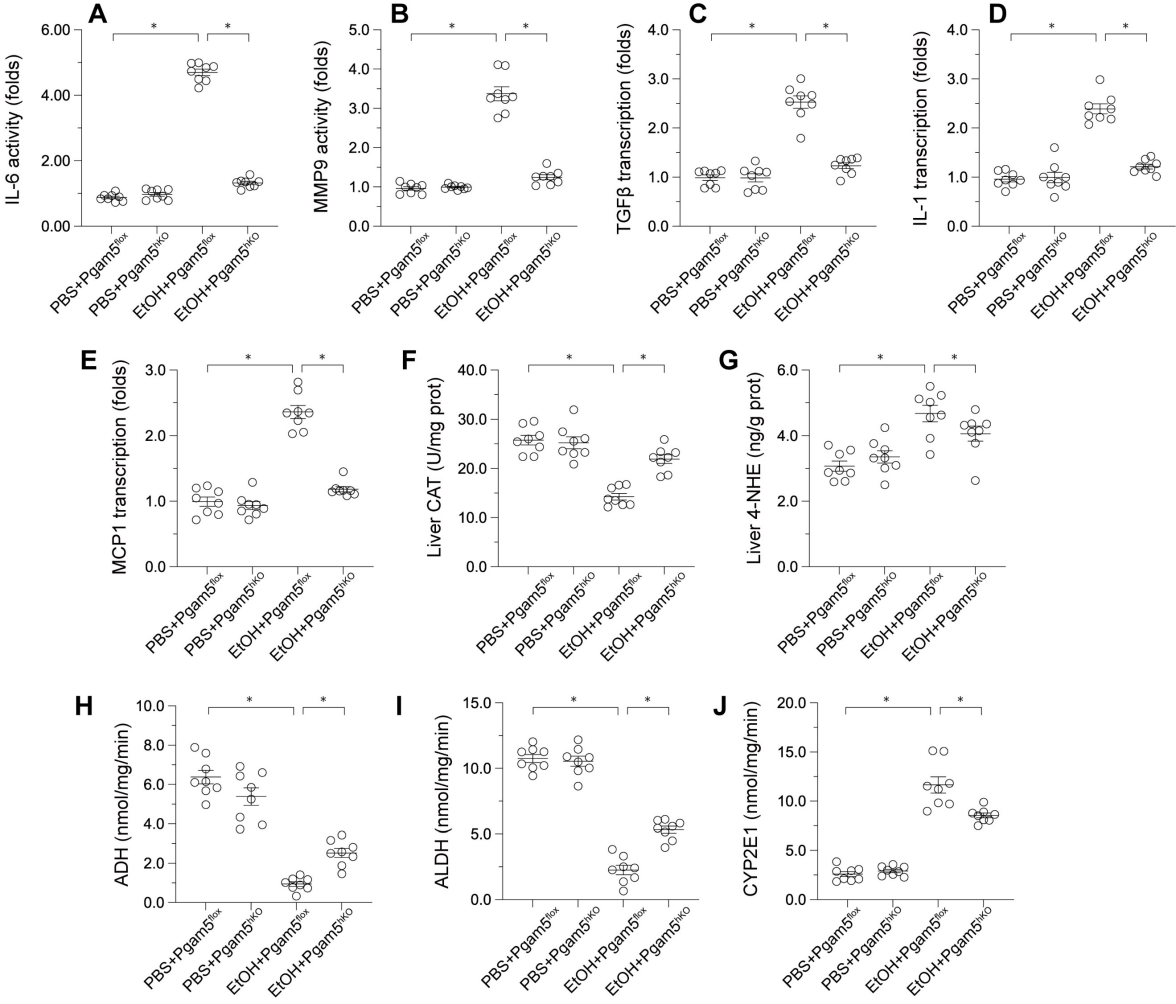
** Pgam5 Deletion Alleviates Alcohol-Induced Hepatic Inflammation, Lipid Peroxidation, and Metabolic Disorder.** *Pgam5*-knock out (*Pgam5^hKO^*), and *Pgam5^f/f^* mice were fed with the liquid control (pair-fed) or ethanol diet (ethanol-fed) (5% ethanol) for 8 weeks. **A-B.** ELISA kits were used to analyze the activities of IL-6 and MMP9. **C-E.** RNA was isolated from liver tissues and the transcription of MCP1, TGFβ, and IL-1 was determined by qPCR**. F-J.** ELISA kits were used to evaluate the activities of CAT, 4-NHE, ADH, ALDH and CYP2E1 in liver tissues. *p<0.05.

Pathological factors such as inflammation, oxidative stress, and altered alcohol metabolism are crucial in ALD progression. ELISA assays demonstrated that MMP9 and IL-6 activities were significantly elevated in male *Pgam5^f/f^* mice and reduced in *Pgam5^hKO^* mice (Figures 5A-B). Correspondingly, the transcription of pro-inflammatory genes (Tgfβ, Il-1, Mcp1) escalated in response to EtOH in *Pgam5^f/f^* mice but was mitigated following *Pgam5* knockout (Figures 5C-E). Liver catalase (CAT) content decreased and 4-Hydroxynonenal (4-HNE) content increased in male *Pgam5^flf^* mice, indicative of oxidative stress, while these changes were not evident in *Pgam5^hKO^* mice (Figures 5F-G). Furthermore, alcohol dehydrogenase (ADH) and aldehyde dehydrogenase (ALDH) activities were compromised in male *Pgam5^hKO^* mice, alongside increased CYP2E1 activity (Figures 5H-J). Conversely, *Pgam5* loss prevented these alterations in ADH and ALDH activities and maintained CYP2E1 levels in the ALD model. Overall, these data suggested that Pgam5 deficiency alleviates alcohol-mediated hepatic inflammation, lipid peroxidation and metabolic disorder.

## Discussion

Alcoholic liver disease (ALD), a major cause of morbidity and mortality globally, is characterized by a spectrum of liver pathologies precipitated by excessive alcohol consumption. Central to the pathogenesis of ALD is mitochondrial dysfunction, which is implicated in the onset and progression of liver damage 43, 44. Our study elucidates the roles of Pgam5 and VDAC1 in mitochondrial homeostasis under the duress of alcohol-induced stress, offering novel insights into their mechanistic interplay in ALD.

Pgam5, traditionally associated with mitochondrial dynamics and apoptosis, emerges as a key modulator in ethanol-induced hepatocyte dysfunction. Our findings demonstrate that Pgam5 silencing significantly attenuates hepatocyte apoptosis and preserves cell viability in the presence of ethanol. This aligns with existing literature that posits a crucial role for Pgam5 in mitochondrial quality control and cell survival under stress conditions 25. The mechanistic underpinnings revealed in our study, particularly the role of Pgam5 in exacerbating ethanol-induced hepatocyte dysfunction, underscore its potential as a therapeutic target in ALD.

### VDAC1 Oligomerization and Mitochondrial Integrity

VDAC1, integral to mitochondrial permeability and apoptosis, was found to oligomerize in response to ethanol exposure, triggering mitochondrial dysfunction. Our study uniquely links Pgam5 to the regulation of VDAC1 oligomerization, a process crucial for maintaining mitochondrial integrity. The inhibition of VDAC1 oligomerization via Pgam5 silencing or pharmacological intervention (VBIT-12) resulted in the preservation of mitochondrial function, as evidenced by the maintenance of mitochondrial membrane potential and reduced ROS production. This finding provides a deeper understanding of the molecular cascades involved in mitochondrial dysregulation in ALD.

The interplay between Pgam5 and VDAC1, as demonstrated in our study, suggests a complex regulatory mechanism influencing mitochondrial homeostasis in ALD. The Pgam5-induced VDAC1 oligomerization appears to be a pivotal event leading to mitochondrial dysfunction, highlighting a novel pathway that could be exploited for therapeutic intervention. This interaction could be a part of a larger network of mitochondrial regulatory mechanisms, which warrants further investigation to fully elucidate the pathways involved in ALD pathophysiology.

The implications of our findings extend beyond the academic exploration of ALD pathogenesis, offering potential clinical applications. Targeting Pgam5 or VDAC1, either through genetic manipulation or pharmacological inhibition, could emerge as a viable therapeutic strategy to mitigate mitochondrial dysfunction in ALD. However, translating these findings into clinical practice necessitates extensive research, including preclinical and clinical trials, to ascertain the efficacy and safety of such interventions.

While our study provides valuable insights, it is not without limitations. The use of L02 cells and animal models, though informative, may not fully replicate the complexity of ALD in humans. Future research should focus on validating these findings in human studies and exploring the broader implications of Pgam5 and VDAC1 modulation in liver diseases. Additionally, investigating the interaction of these proteins with other signaling pathways could unveil more comprehensive therapeutic targets.

In conclusion, our study highlights the critical roles of Pgam5 and VDAC1 in the context of ALD, particularly their involvement in mitochondrial dysfunction. By elucidating these molecular mechanisms, we pave the way for novel therapeutic strategies aimed at mitigating the deleterious effects of alcohol on the liver. The potential of targeting mitochondrial pathways in ALD represents a promising frontier in the quest to alleviate the burden of this disease.
